# Sex differences in hippocampal β-adrenergic receptor subtypes drive retrieval, retention, and learning of cocaine-associated memories

**DOI:** 10.3389/fnbeh.2024.1379866

**Published:** 2024-05-14

**Authors:** Melanie M. Berry, Beau Miller, Silvia Kelsen, Carlee Cockrell, Amy Stave Kohtz

**Affiliations:** ^1^Department of Psychiatry, Division of Neurobiology and Human Behavior, University of Mississippi Medical Center, Jackson, MS, United States; ^2^Department of Biological Sciences, Mississippi College, Jackson, MS, United States; ^3^Center for Innovation and Discovery in Addictions, University of Mississippi Medical Center, Jackson, MS, United States

**Keywords:** β-adrenergic receptors, sex differences, drug associated memories, cocaine conditioned place preference, dorsal hippocampus

## Abstract

**Background:**

Drug seeking behavior occurs in response to environmental contexts and drug-associated cues. The presence of these pervasive stimuli impedes abstinence success. β-adrenergic receptors (β-ARs) have a long-standing historical implication in driving processes associated with contextual memories, including drug-associated memories in substance use disorders. However, sex differences in the role of β-adrenergic receptors in drug memories remain unknown.

**Hypothesis:**

Prior reports indicate a selective role for β2-ARs in retrieval and retention of contextual drug memories in males, and substantial sex differences exist in the expression of β-ARs of male and female rats. Therefore, we hypothesized that there are sex differences in selective recruitment of β-ARs during different stages of memory encoding and retrieval.

**Methods:**

The role of β-ARs in driving retrieval and learning of contextual cocaine memories was investigated using cocaine conditioned place preference (CPP) in adult male and female Sprague–Dawley rats. Rats were infused directly to the dorsal hippocampus with Propranolol (β1 and β2) or ICI-118,551 (β1) and/or Betaxolol (β2), immediately prior to testing (retrieval), or paired to each cocaine (10 mg/kp, IP) conditioning session (learning).

**Results:**

In males, administration of either β1, β2, or combined β1 and β2-ARs before the initial CPP testing reduced the expression of a CPP compared to vehicle administration. In females, β2-ARs transiently decreased CPP memories, whereas β1 had long lasting but not immediate effects to decrease CPP memories. Additionally, β1 and combined β1 and β2-ARs had immediate and persistent effects to decrease CPP memory expression. DG Fos + neurons predicted cocaine CPP expression in males, whereas CA1 and CA3 Fos + neurons predicted cocaine CPP expression in females.

**Conclusion:**

There are significant sex differences in the role of dorsal hippocampus β-ARs in the encoding and expression of cocaine conditioned place preference. Furthermore, sub regions of the dorsal hippocampus appear to activate differently between male and female rats during CPP. Therefore DG, CA3, and CA1 may have separate region- and sex-specific impacts on driving drug- associated, or context-associated cues.

## 1 Introduction

Sex differences are found in the progression of substance use disorders. Men and women differ in the initiation, progression, and relapse to cocaine use. Women show higher response rates to triggers of cravings and use of cocaine (Kennedy et al., 2013), develop cocaine dependence more rapidly (Becker and Koob, 2016; Castro-Zavala et al., 2020), differ in response to social cues (Waldrop et al., 2010) and seek treatment options sooner than do men (Lynch et al., 2002). These observations are well recapitulated in rodent models (Becker and Hu, 2008; Fattore et al., 2008; Anker and Carroll, 2011). Thus, understanding mechanisms driving sex differences in addiction is important in the pursuit of addiction therapies.

The environment in which a drug was taken can incentivize drug craving and contribute to relapse (Childress et al., 1988). Memory retrieval following the presentation of environmental cues requires signaling in the dorsal hippocampus (Matus-Amat, 2004; Wiltgen et al., 2010; Ferrara et al., 2019; Anderson and Floresco, 2021). Prior studies indicate inhibition of the dorsal hippocampus during condition place preference testing (CPP) (Meyers et al., 2006; Hitchcock and Lattal, 2018), attenuates the retention, consolidation, and retrieval of drug-associated memories (Fuchs et al., 2005; Crombag et al., 2008). We have previously implicated the dorsal hippocampus as a focal point that may drive sex differences in cocaine use disorder, as antagonizing noradrenergic or serotonergic receptors in the dorsal hippocampus can attenuate operant cocaine-seeking behavior in a sex dependent manner (Kohtz and Aston-Jones, 2017). Thus, the dorsal hippocampus drives contextual cue driven cocaine seeking and may contribute to sex differences in context dependent relapse.

Cocaine-associated memories are maintained by the activation of the β-adrenergic receptor (β-AR), as these memories can evoke drug craving and reinstatement. Previously, it has been shown that blocking β1-ARs, but not β2-ARs in male rats can induce a deficit in cocaine associated memory retrieval and retention of CPP (Otis et al., 2013; Fitzgerald et al., 2016). Additionally, evidence reveals that preventing the retrieval of drug associated memories with β-adrenergic receptor (β-AR) antagonists causes long-lasting impairments in retrieval in rodents (Otis and Mueller, 2011; Otis et al., 2013). This retrieval prevention has been shown to provide protection against drug-induced reinstatement (Otis and Mueller, 2011), confirming that disruption of retrieval would limit relapse susceptibility. However, sex differences in the selective role of these receptors in driving drug memories remain unknown. Herein, we investigated the hypothesis that there are sex differences in the role of dorsal hippocampus β-adrenergic receptors in cocaine-associated contextual memories using CPP.

## 2 Methods

### 2.1 Animals

Female (200–250 g, *n* = 103) and male (275–325 g, *n =* 115) Sprague Dawley rats (Envigo) were singly housed under a reversed 12 h/12 h light/dark cycle (lights off at 0800 h) per prior reported methods (Zakharova et al., 2009; Turner et al., 2014; Venniro et al., 2018; Lorenz et al., 2022). All experiments were conducted between 10 am and 2 pm, Monday through Friday. Rats had free access to food and water and were housed in the animal facility at the University of Mississippi Medical Center. All experiments were approved by the institutional animal care and use committee (IACUC # 2022–1,170) and conducted in accordance with the National Institutes of Health specifications outlined in their Guide for the Care and Use of Laboratory Animals.

### 2.2 Dorsal hippocampus cannula implantation

**Figure 1:**
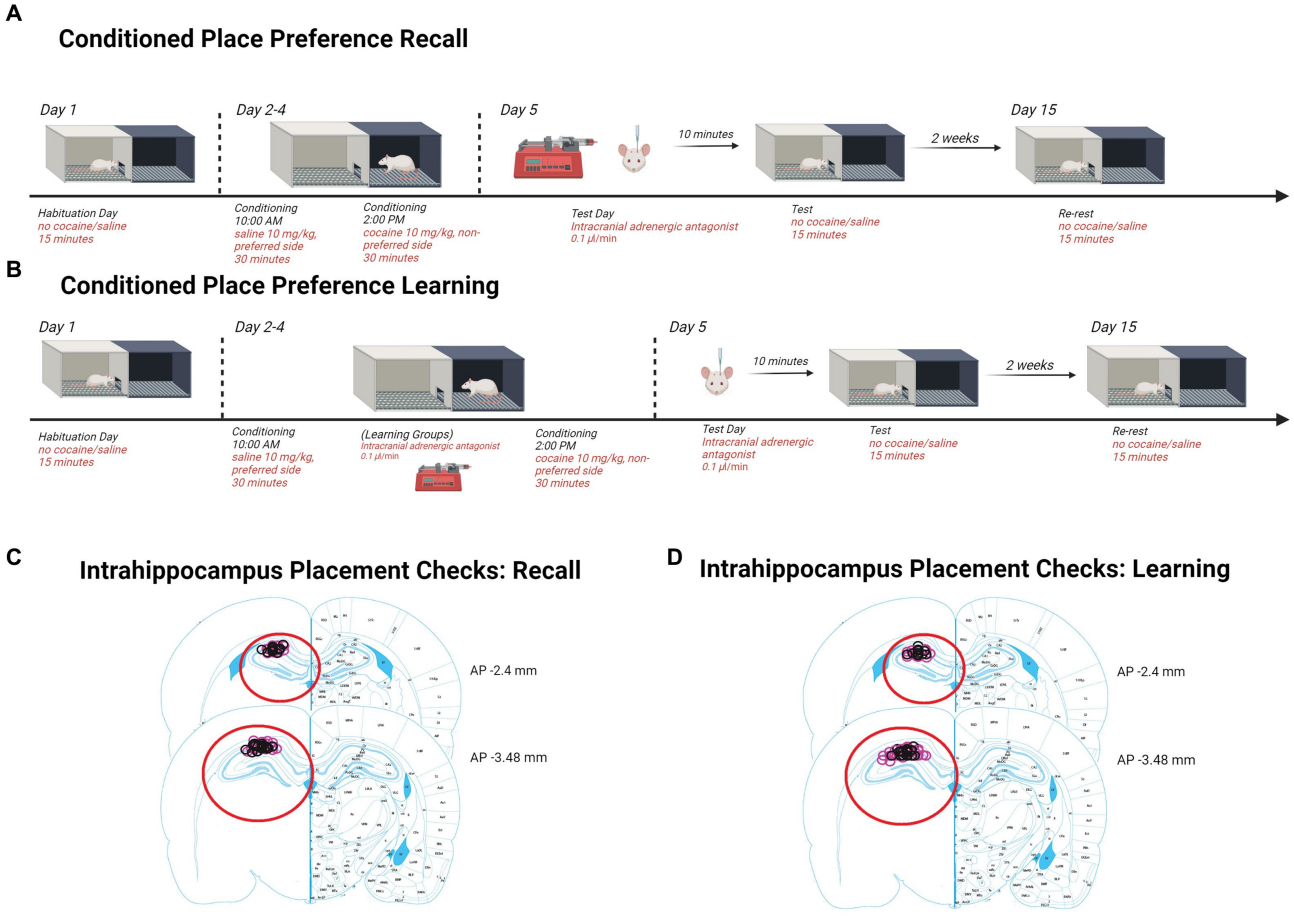
Schematics and placement checks in all experiments. **(A)** Diagram depicting methods for testing retrieval and retention in CPP. Rats are first assessed for side preferences on day 1 for 15 min. Days 2–4, rats received saline in the AM and were placed in their preferred side and cocaine in the PM and were placed in their non-preferred side. Day 5, rats received β-adrenergic receptor antagonists intrahippocampally 10 min prior to being tested for 15 min. Two weeks later, rats were re-tested for 15 min to assess retention. Histological confirmation of intra-hippocampal infusion sites. **(B)** Diagram depicting methods for testing learning and retention in CPP. Rats chose a preference side on day 1 during a 15-min interval. Days 2–4, rats received saline in the AM and were placed in their preferred side. In the PM, rats were intrahippocampally injected 10 min prior to receiving cocaine and were placed in their non-preferred side. On day 5, females were tested for 15 min. Two weeks later, females were re-tested for 15 min to assess consolidation retention. Histological confirmation of intra-hippocampal infusion sites. **(C)** Injection sites in recall experiments are indicated by black (male) and pink (female) circles on the right graph (adapted from Paxinos and Watson, 2014). **(D)** Injection sites in learning experiments are indicated by black (male) and pink (female) circles on the right graph (adapted from Paxinos and Watson, 2014).Created with Biorender.com.

**Figure 2:**
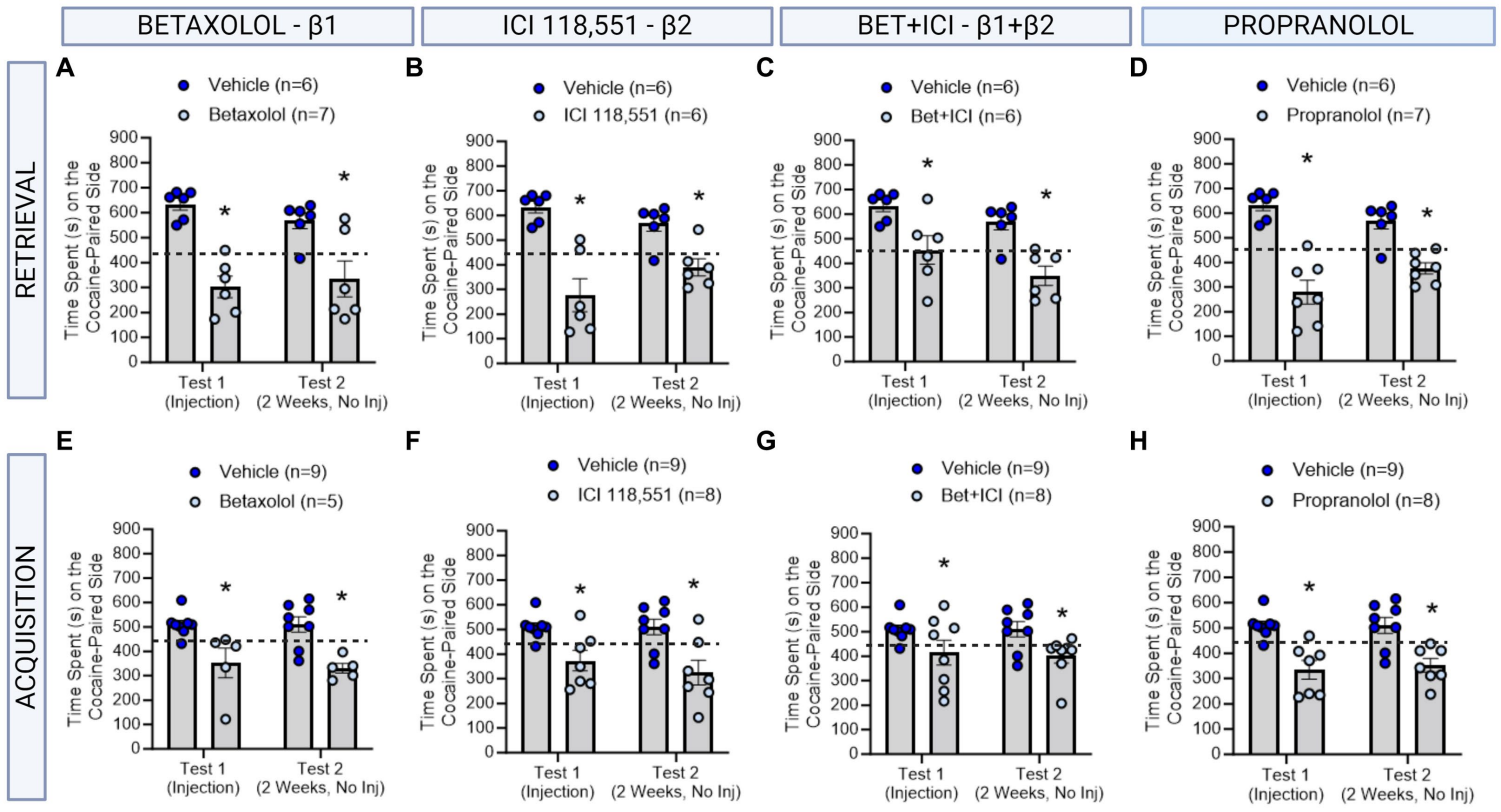
The role of β-ARs in CPP memories in males. **(A–D)** Effects of adrenergic receptor antagonists injected immediately prior to retrieval in males. Betaxolol **(A)**, ICI 118,551 **(B)**, Betaxolol + ICI 118,551 **(C)** and Propranolol **(D)** had long-lasting effects to decrease CPP memories. *indicates *p* < 0.05. **(E–H)** Effects of adrenergic receptor antagonists injected immediately prior to each cocaine conditioning session in males. Betaxolol **(E)**, ICI 118,551 **(F)**, Betaxolol + ICI 118,551 **(G)** and Propranolol **(H)** had immediate and persistent effects to decrease CPP memory expression. * indicates *p* < 0.05 compared to aCSF vehicle.

Rats were anesthetized by intraperitoneal (i.p.) injection of ketamine/xylazine (56.5/8.7 mg/kg) and given carprofen (1 mg/kg) as an analgesic. Bilateral guide cannulas were implanted to the dorsal hippocampus (AP: −3.0, ML: ±2.0, DV: −2.5). Cannula placement was confirmed for each rat as indicated in the corresponding figures (Retrieval; Figure 1, Learning; Figure 2).

### 2.3 Drugs

Cocaine hydrochloride (NIDA, Research Triangle Park, NC) was dissolved in 0.9% sterile saline and was administered intraperitoneally (i.p.) at a dose of 10 mg/kg immediately before being placed in the conditioning chamber. Betaxolol (*β*1 AR antagonist, 1 nmol/1.0 *μ*L/side, Betaxolol) and/or ICI 118551, (*β*2 AR antagonist, 1 nmol/1.0 *μ*L/side, ICI 118551), or S/R-propranolol (*β*1+ *β2* AR antagonist, 1 nmol/1.0 *μ*L/side, Propranolol; combined *β*-adrenergic and 5-HT1A/B receptor antagonist; Pazos et al., 1985), were intracranially administered at a rate of 0.1 *μ*L/min 10 min before each cocaine conditioning session (learned) or before each testing session (retrieval). All intracranially administered drugs were dissolved in aCSF obtained from Tocris Sciences. In addition to the selective β-adrenergic receptor antagonists, S/R-Propranolol at 10 mg/kg (5 mg/mL R /5 mg/mL S) was also tested given its widespread clinical and pre-clinical use (Schwabe et al., 2012; Haubrich et al., 2020; Chen et al., 2021).

### 2.4 Conditioned place preference

#### 2.4.1 Chamber

Four two-compartment chambers automatic door CPP boxes were used for habituation, testing, and conditioning. The two chambers measured (12″ x 8.25″ x 8.25″) and were separated by a center piece containing an automatic guillotine door. The black and white sides both had grid rod floors. During conditioning, rats were isolated to either the white side or the black side. During habituation and tests days, rats had access to the entire apparatus with the guillotine door held open.

#### 2.4.2 Habituation

On day 1 at 12 pm, rats were placed in the CPP box on the side with the black chamber with the guillotine door open giving access to both sides for 15 min of habituation as seen in Figures 1, 2. Rats with an observable side preference (>60% of the time spent on one side or the other) during habituation were counter-conditioned with cocaine on the non-preferred side.

#### 2.4.3 Conditioning

Starting on day 2, rats were placed in CPP boxes at 10 am and 4 h later at 2 pm. The 10 am session consisted of the rats receiving saline injections intraperitonially immediately before being placed in the chamber that was preferred on habituation day. The rats were confined to this side for 30 min with the guillotine door closed before being removed and placed back in their home cage (Figure 1). At 2 pm, 4 h later, rats received cocaine injections intraperitonially immediately before being placed in the chamber that was not initially preferred on habituation day. The rats were confined to the non-preferred side for 30 min with the guillotine door closed before being removed and placed back in their home cage (Figure 1).

#### 2.4.4 Retrieval testing

To test whether Betaxolol, and/or ICI 118,551, or Propranolol treatment would prevent retrieval of cocaine seeking behavior on ED1, each group of rats were intracranially administered one of the drugs at a rate of 0.1 *μ*L/min 10 min before testing in the CPP chamber with the guillotine door opened. Following this test, the rats were returned to their home cages for 2 weeks (Figure 1).

#### 2.4.5 Learning

In a separate group of rats, we tested the effects of β-ARs on acquisition/consolidation (learning) of CPP memory. To test the effects of β1 and β2 adrenergic receptor antagonists on the expression of CPP memories, each rat was intracranially administered one of the drugs at a rate of 0.1 *μ*L/min 10 min before each cocaine injection intraperitonially and were placed in their non-preferred side on conditioning days (Figure 2).

#### 2.4.6 Retention testing

To test the persistence of β-adrenergic receptor antagonists’ effects on the expression of CPP memories, all rats were re-tested 2 weeks later with no injections given (Figure 2).

### 2.5 Transcardial perfusion

Prior to perfusions, rats were anesthetized using isoflurane and placed on a surgical plane. Two 3-4in incisions were made through the abdominal wall and cutting through the rib cage. The sternum was clamped with the hemostat and a 15-gage blunt-tipped perfusion needle was inserted into the ascending aorta and secured with a hemostat. Finally, we made an incision to the animal’s right atrium using iris scissors to create as large an outlet as possible without damaging the descending aorta. Rats were perfused with 1 mL/gram body weight of 1x PBS, followed by 4% paraformaldehyde (PFA) at a constant speed of ~1 mL/5 s. Brains were post-fixed in 4% PFA for 24 h at 4 degrees Celsius, before transfer to 30% sucrose cryoprotectant and sectioning.

### 2.6 Immunohistochemistry

Immunohistochemistry was implemented as previously described (Kohtz and Aston-Jones, 2017). Sections were blocked in 10% normal donkey serum (Jackson Labs) for 2 h in PBS-T with 0.1%bAz. Rabbit anti-Fos antibody (1: 1000, Synaptic Systems). The sections were then rinsed and incubated in secondary (Alexa fluor 488; 1: 500, Invitrogen) for 2 h. Sections were then mounted and imaged on a Zeiss Axiozoom x16.

## 3 Results

### 3.1 A single infusion of an antagonist targeting β1 and/or β2 ARs impaired cocaine contextual memory retrieval and retention in male rats

**Figure 3:**
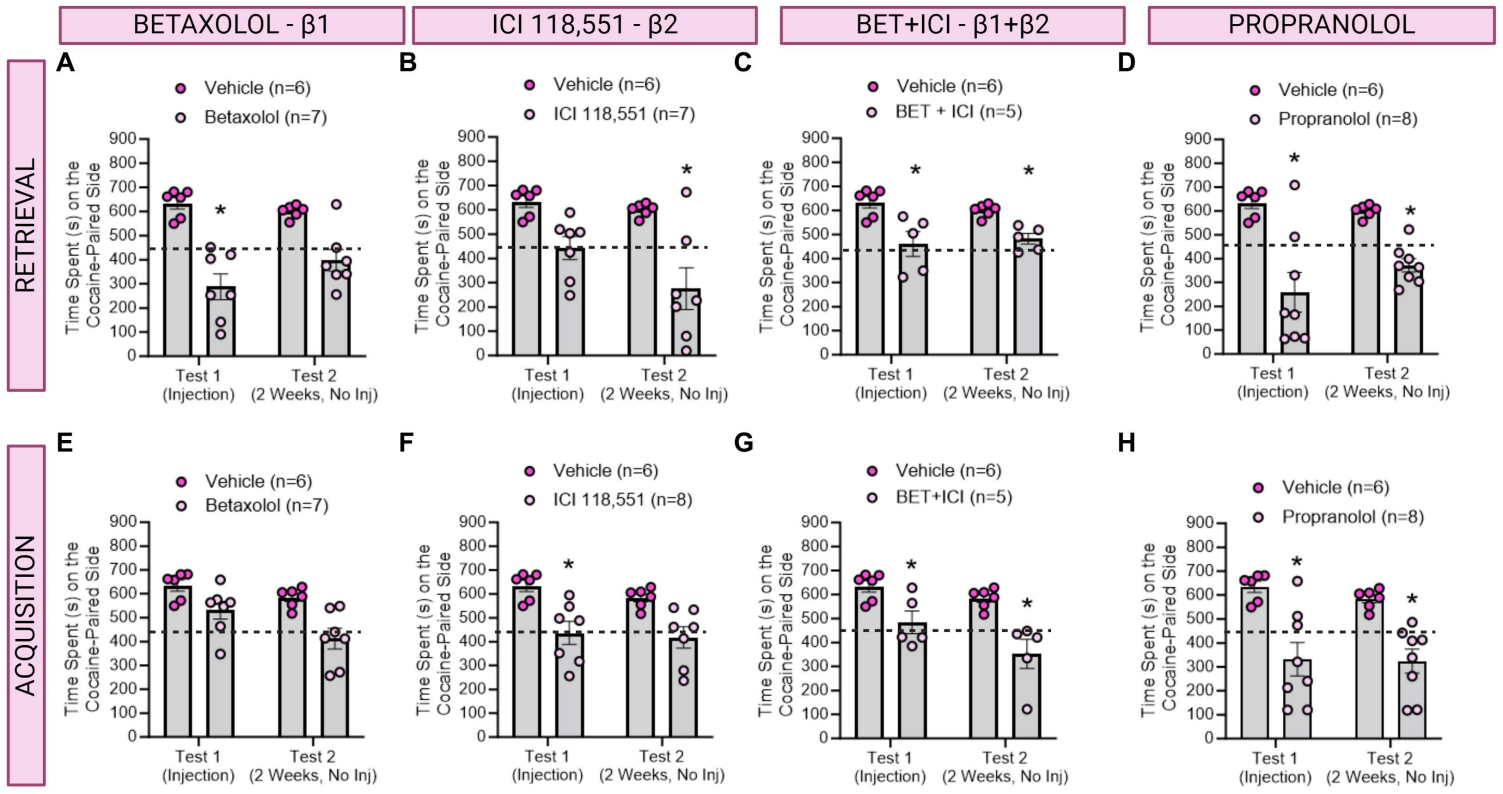
The role of β-ARs in CPP memories in females. **(A–D)** Effects of adrenergic receptor antagonists injected immediately prior to retrieval in females. **(A)** Betaxolol transiently decreased CPP memories, whereas ICI 118,551 **(B)** had long-lasting, but not immediate effects to decrease CPP memories. Betaxolol + ICI 118,551 **(C)** and Propranolol **(D)** impaired CPP memory retrieval and retention tested 2 weeks later. * indicates *p* < 0.05. **(E–H)** Effects of adrenergic receptor antagonists injected immediately prior to each cocaine conditioning session in females. ICI 118,551 **(F)**, Betaxolol + ICI 118,551 **(G)**, and Propranolol **(H)** had immediate and persistent effects to decrease CPP memory expression. *indicates *p* < 0.05 compared to aCSF vehicle.

We first tested the effects of administering a single injection of a β-adrenergic receptor antagonists 10 min prior to testing for cocaine conditioned place preference memory retrieval in male rats (*n* = 6-7/group) using the timeline described in Figure 1. Using a repeated measures two-way ANOVA, we found a significant interaction between β-adrenergic receptor antagonists and test day [*F*(4,26) = 3.176, ηp^2^ = 0.33, *p* = 0.0299] and a significant main effect of drug condition independent of test day [*F*(4,26) = 9.833, ηp^2^ = 0.60, *p* < 0.0001]. Post-hoc analyses reveal that Betaxolol (β1-AR) administered prior to testing, impaired retrieval of the CPP memory on test 1, and impaired retention when tested on test 2 [*F*(1,10) = 26.59, *p* = 0.0004; Figure 3A]. ICI 118,551 (β2-AR) administered prior to testing, impaired retrieval of the CPP memory on test 1, and impaired retention when tested on test 2 [*F*(1,10) = 30.53, *p* = 0.0003; Figure 3B]. When combined, Betaxolol + ICI 118,551 were less effective than when administered alone, however similarly impaired retrieval and retention of a CPP memory as when administered alone [*F*(1,10) = 18.44, *p* = 0.0016; Figure 3C], as did Propranolol [*F*(1,11) = 86.35, *p* = 0.0002; Figure 3D]. These effects suggest that the efficacy of β-adrenergic receptor antagonists to attenuate the retrieval and retention of cocaine memories remained consistent across receptor subtypes, when administered directly to the dorsal hippocampus.

### 3.2 Infusions of antagonists targeting β1 and/or β2 ARs impaired cocaine contextual memory learning and retention in male rats

We next tested the effects of administering an infusion of a β-adrenergic receptor antagonists 10 min prior to each cocaine conditioning session in male rats (n = 5-8/group) using the timeline described in Figure 2. We found a significant main effect of treatment to decrease cocaine preference persistently in male rats [*F*(4,30) = 5.626, ηp^2^ = 0.43, *p* = 0.0017]. Betaxolol (β1-AR antagonist) [*F*(1,11) = 10.31, *p* = 0.0083 Figure 3E], ICI 118,551 (β2-AR antagonist; F (1,11) = 26.06, *p* = 0.0003; Figure 3F), Betaxolol + ICI 118,551 [*F*(1,14) = 7.046, *p* = 0.0189, Figure 3G], and Propranolol [β1 + β2 AR antagonist; *F* (1,13) = 20.83, *p* = 0.0005; Figure 3H] to persistently decrease the expression of the CPP memory. These effects suggest that the efficacy of β-adrenergic receptor antagonists to attenuate learning persistently of cocaine memories remained consistent across receptor subtypes, when administered directly to the dorsal hippocampus.

### 3.3 A single infusion of an antagonist targeting β1 ARs impaired retrieval, whereas antagonists to β2 ARs impaired retention, of contextual cocaine associated memories in female rats

**Figure 4:**
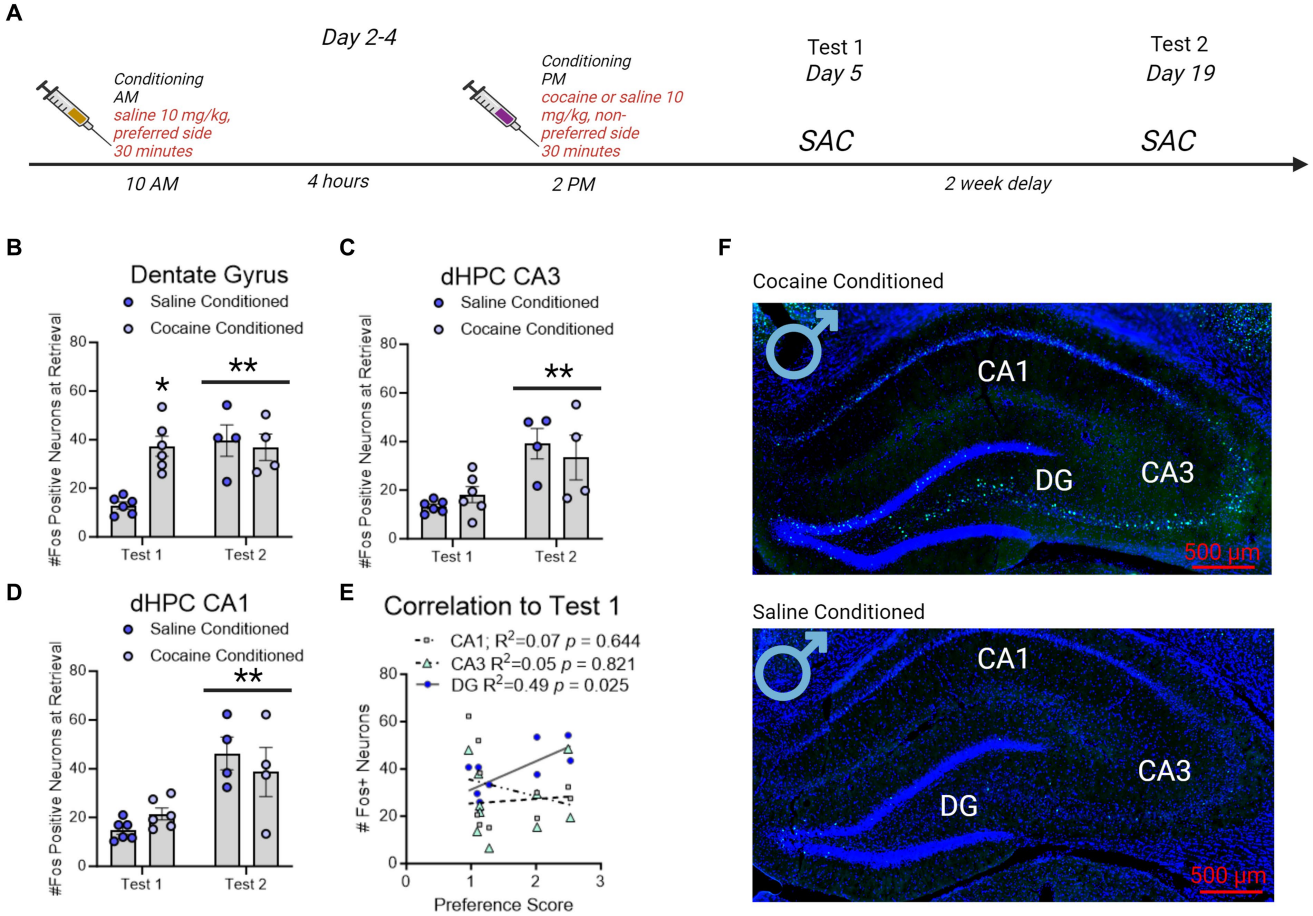
Fos + neuron reactivity in subregions of the dorsal hippocampus following CPP testing in male rats. **(A)** Timeline of CPP and sacrifice conditions. Rats were sacrificed 60 min after the conclusion of either the first or second CPP test. **(B)** Fos + neurons in the dentate gyrus increased with cocaine-conditioning compared to saline-conditioned controls. * indicates *p* < 0.05. Neurons in the dentate gyrus showed increased Fos + expression on test 2 independent of saline or cocaine administration. ** indicates *p* < 0.05. **(C,D)** The number of Fos + neurons in CA3 **(C)** and CA1 **(D)** did not increase with cocaine conditioning. Neurons in CA3 and CA1 did show increased Fos + expression on test 2 independent of saline or cocaine administration. **indicates *p* < 0.05. **(E)** Correlation matrix representing R^2^ values in male rats. Test 1 CPP memory expression correlated to test 2 memory expression, and to dentate gyrus (DG), but not CA3 or CA1, Fos + neurons in the dHPC. Red underline indicates *p* < 0.05. **(F)** Example images of cocaine conditioned (top) and saline conditioned (bottom) males sacrificed on test 1. Fos + neurons are represented in green and Dapi (nuclei) in blue.

We then investigated the effects of administering a single injection of a β-adrenergic receptor antagonists 10 min prior to testing for cocaine conditioned place preference memory expression in female rats (*n* = 5-8/group) per the timeline depicted in Figure 1. We found a significant main effect of treatment to decrease CPP memory expression retrieval and retention in female rats [*F*(4,28) = 9.590, ηp^2^ = 0.58, *p* < 0.0001]. Post-hoc analyses reveal Betaxolol transiently decreased CPP expression [*F*(1,10) = 26.59, *p* = 0.0004; Figure 4A], whereas ICI 118,551 had long-term, but not immediate, effects to attenuate cocaine CPP [*F*(1,10) = 30.53, *p* = 0.0003; Figure 4B]. Combined antagonists Betaxolol + ICI 118,551 [*F*(1,9) = 24.16, *p* = 0.0008, Figure 4C] and Propranolol [*F*(1,11) = 86.35, *p* = 0.0002; Figure 4D] had both immediate and sustained effects to attenuate cocaine CPP expression. Therefore, in female rats, β1-ARs likely drive recall, whereas β2-ARs drive retention, of cocaine CPP memories.

### 3.4 Infusions of antagonists targeting both β1 and β2 receptors impaired learning of contextual cocaine memories in female rats

In a separate group of rats, we investigated the effects of β-adrenergic receptor antagonists on CPP learning in female rats (*n* = 5-8/group) per the timeline in Figure 2. There was a main effect of drug treatment to decrease expression of cocaine preference [*F*(4,28) = 6.512, ηp^2^ = 0.48, *p* = 0.0008; Figure 4]. *Post-hoc* analysis revealed Betaxolol [*F*(1,11) = 10.31, *p* = 0.0083; Figure 4E], ICI 118,551 [*F*(1,11) = 26.06, *p* = 0.0003; Figure 4F], Betaxolol + ICI 118,551 (F(1,9) = 23.53, *p* = 0.0009; Figure 4E) and Propranolol [*F*(1,12) = 19.06, *p* = 0.0006; Figure 4E] persistently attenuated cocaine CPP when administered paired with cocaine conditioning. Therefore, impairing recall or retention by β1 and β2 antagonism respectively, is sufficient to impair learning of cocaine CPP in female rats.

### 3.5 Fos + neurons in the dentate gyrus predicted contextual cocaine memories in male rats, whereas Fos + neurons in CA1 and CA3 predicted contextual cocaine memories in female rats

**Figure 5:**
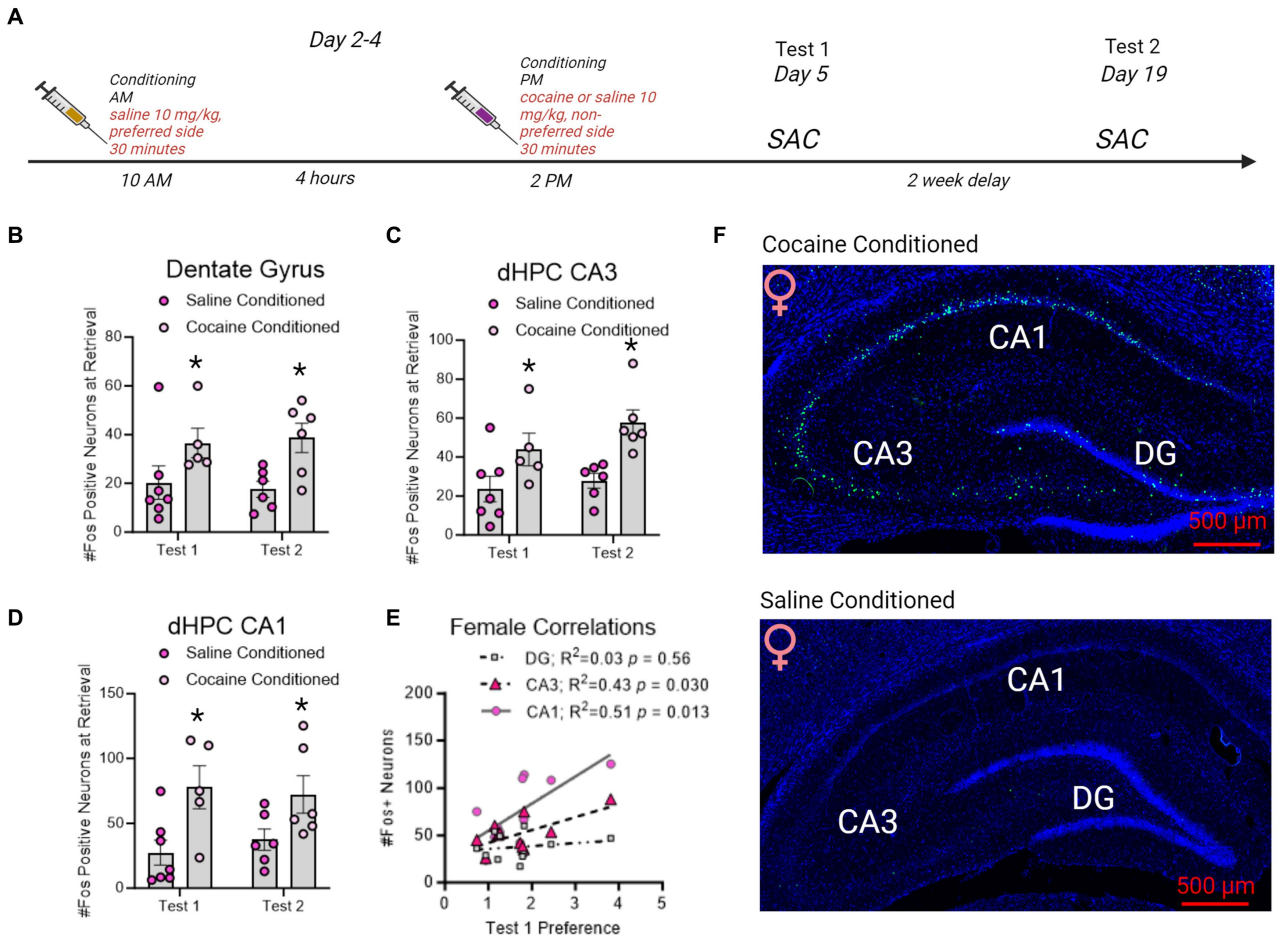
Fos + neuron reactivity in subregions of the dorsal hippocampus following CPP testing in female rats. **(A)** Timeline of CPP conditioning and sacrifice times as in Figure 5. **(B–D)** Fos + neurons in the dentate gyrus, CA3, and CA1 increased with cocaine-conditioning and was retained to test 2. *indicates *p* < 0.05. **(E)** Correlation matrix representing *R*^2^ values in female rats. Test 1 CPP memory expression correlated to CA3 and CA1 Fos + neurons in the dHC. Red underline indicates *p* < 0.05. **(F)** Example images of cocaine conditioned (top) and saline conditioned (bottom) females sacrificed on test 1. Fos is represented in green and Dapi in blue.

A separate group of rats were used to identify regional Fos expression in the dorsal hippocampus during CPP retrieval. Male (*n* = 4/group) and female (*n* = 5-7/group) rats were conditioned for cocaine or saline (controls). Rats were sacrificed 60 min after the conclusion of CPP test and brains were examined for Fos expression in subregions of the dHC (Figure 5A).

There was a significant interaction between test day and conditioning in male rats, wherein cocaine conditioned, but not saline conditioned, rats had increased Fos + neurons in the dentate gyrus on test day 1 [*F*(1,16) = 9.858, *p* = 0.0063] only. Notably, all male rats irrespective of conditioning had elevated Fos + neurons on test day 2 compare to test day 1 [DG: *F*(1,16) = 9.254, *p* = 0.0078 Figure 5B]; CA3: [*F*(1,16) = 17.48, *p* = 0.0007 Figure 5C; CA1: *F*(1,16) = 22.49, *p* = 0.0002; Figure 5D]. The effects of elevated Fos + neurons on test day 2 compared to test day 1 recapitulate those shown in prior reports (Zhou et al., 2013). Furthermore, DG, but not CA3 or CA1, Fos + neuron expression significantly correlated to Test 1 (Figure 5E).

**Figure 6:**
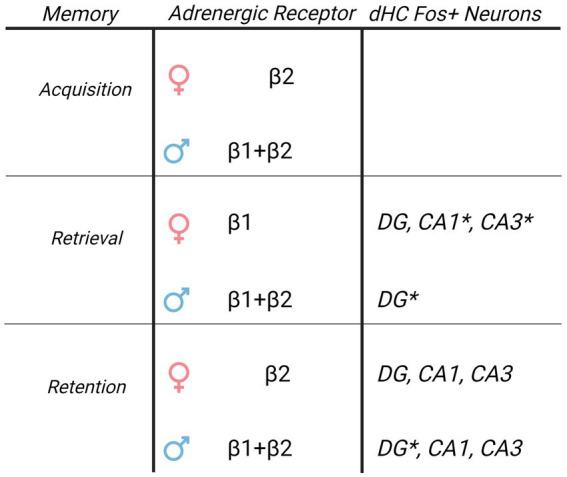
Sex specific roles of β-AR and Fos + immunoreactivity in the dorsal hippocampus driving cocaine conditioned place preference. Table of β-AR receptors and Fos + neuron distribution in dorsal hippocampus CA1 and their involvement in CPP memories based upon current results. While β-ARs have global involvement in driving CPP memories in males, the role of β-ARs in females is restricted to subtype and memory modality. Although elevated Fos + neuron activity following retrieval of CPP memories is similar, the relationship between Fos + neurons and CPP memory expression is sexually-differentiated. DG Fos + neurons predict CPP expression in males, and CA1/CA3 Fos + neurons predict CPP expression in females. * indicates Fos + neurons that correlated to the magnitude of memory expression on test 1 in each sex.

In female rats conditioned with cocaine, Fos + neurons in the dentate gyrus [*F*(1,20) = 9.989, *p* = 0.0049; Figure 6], CA3 [*F*(1,20) = 15.08, *p* = 0.0009; Figure 6], and CA1 [*F*(1,20) = 12.24, *p* = 0.0023; Figure 6] were elevated by CPP retrieval on both test 1 and test 2 compared to rats conditioned with saline alone. With cocaine-conditioning and was retained to test 2. In contrast to male rats, CA1 and CA3 Fos + neurons correlated to test 1 CPP expression, but DG Fos + neurons did not (Figure 6).

## 4 Discussion

Herein, we show sex differences in the role of β-adrenergic receptors in driving contextual cocaine memories. Administration of a singular infusion to the dHC of Betaxolol, ICI 118,551, and Propranolol had long lasting, immediate, and persistent effects to decrease CPP memories in male rats, indicating β1-ARs and β2-ARs similarly impact cocaine CPP of male rats. Our results additionally show that β1-ARs and β2-ARs differentially impact cocaine CPP of female rats. Betaxolol transiently decreased CPP memories, whereas ICI 118,551 had long-lasting, but not immediate effects to decrease CPP memories. Propranolol, a combined β1/β2-adrenergic and 5-HT1A/1B receptor antagonist, both impaired CPP memory retrieval and retention tested 2 weeks later, whereas ICI 118,551 and Propranolol had immediate and persistent effects to decrease CPP memory expression. Together, these findings indicate that there are significant sex differences in the role of dorsal hippocampus β-ARs in the encoding and expression of cocaine conditioned place preference.

The dHC is implicated in context-dependent, but not discrete cue- or drug-dependent, cocaine seeking (Raybuck and Lattal, 2014). Underlying sex differences in dHC LTP, spine morphology, and stimulus sensitivity, may drive selective responding of CA1/CA3 to conditioning (Reviewed in Yagi and Galea, 2018). In particular, male rats have greater CA1/CA3 Fos + neuron reactivity during fear conditioning tasks compared to females (Keiser et al., 2017; Colon and Poulos, 2020), which may be driven by greater early and late LTP in CA1/CA3 following high frequency stimuli in males (Yang et al., 2004). Conversely, during operant cocaine memory retrieval, females show a greater increase in CA1/CA3 Fos + neurons compared to males (Kohtz and Aston-Jones, 2017). These effects may be due to sex differences in spine morphology, as female rats show greater population spikes (Woolley et al., 1990), and spine densities (Galea et al., 1997) in CA1/CA3, an effect that may be driven by estradiol (Woolley et al., 1990). Furthermore, previous studies have shown sexually dimorphic Fos expressions in the dHC following CPP studies with female rats showing higher Fos expression than male rats in the CA1 and CA3 (Zhou et al., 2013). Our data confirm and extend these findings, as we show CPP memories are predicted by Fos + neurons in CA1/CA3 in females, but not in males. As is suggested throughout these studies, the profile of hippocampal plasticity and retrieval in response to the context is both sex and *stimuli* dependent; in that females may show greater neuroplasticity in response to contextual drug cues than do males.

Prior studies show distinct mechanisms for β-AR receptor retrieval and reconsolidation of drug-associated contextual memories. β-AR receptor antagonism, in particular to the prelimbic medial prefrontal cortex, but not to the basolateral amygdala, can attenuate cocaine CPP persistently (Otis et al., 2013). Furthermore, they show these effects are driven by limited neuron excitability leading to long-lasting memory impairment and synaptic depression (Otis et al., 2017, 2018), an effect not observed in the basolateral amygdala. Herein, β-AR receptor antagonists applied to the dHC (i.e., onboard during context retrieval), similarly decreased the expression of drug memories persistently, suggesting a similar mechanism of action in the dHC. Further studies are required to investigate if sex-differences in the behavioral response to selective β-AR blockade reflect sex differences in NE-induced potentiation, or neuron excitability.

In addition to β1 and β2 adrenergic receptors being pharmacologically different, it is well established that there are sex differences in β-AR levels and responsiveness between male and female rodents in the hippocampus (Fuchs et al., 2005; Becker and Hu, 2008; Bangasser et al., 2012; Fitzgerald et al., 2016). Previous studies (Fitzgerald et al., 2016) tested the effects of β1 and β2 adrenergic receptor antagonists in male rats found selective differences; however, β-AR receptor antagonists were administered systemically. Higher doses of β1 (10 mg/kg and 20 mg/kg) receptor antagonists showed an effect whereas lower doses (3 mg/kg) only attenuated CPP long term, but not immediately. Additionally, β2 receptor antagonists at lower doses (4 mg/kg and 8 mg/kg) did not reduce initial expression of CPP in previous studies. These effects are in contrast to ours, as we show using an intrahippocampal approach, β1 and β2 receptor antagonists similarly impact CPP in male rats, both transiently and persistently. Although there is evidence of central effects with systemic administration (Uchida, et al., 2002; Hare et al., 2006), both Betaxolol and ICI-118,551 are lipophilic molecules, the differences in the observed effects might be contributable to their efficacy in passing the blood brain barrier as compared to a direct intracranial infusion.

It has previously been found that Propranolol was effective in animal studies to disrupt anxiety caused by withdrawal and associated drug seeking in male rats (Harris and Aston-Jones, 1993a,b; Smith and Aston-Jones, 2008). Additionally, Propranolol and carvedilol have shown promising effects in clinical studies to reduce cocaine-seeking behaviors (Sofuoglu, 2000; Kampman, 2009). While these drugs are typically used as *β*-adrenergic receptor antagonists, Propranolol is also a 5-HT1A/1B receptor antagonist (Pazos et al., 1985). It has been previously found by our lab that 5-HT signaling is involved in drug reward (Harris et al., 2001; Harris and Aston-Jones, 2001), and we hypothesize that some of the effects of Propranolol to influence drug seeking may also involve antagonism of 5-HT receptors. In the following study, S-Propranolol, an adrenergic and serotonin receptor antagonist, and its enantiomer R-Propranolol that antagonizes 5-HT receptors, Previous research has found that in male rats, Betaxolol treated rats were less likely to show a CPP than saline-treated rats. ICI 118,551 treated rats were equally likely to show a CPP for retrieval, but only a higher dose (8 mg/kg) treatment of ICI 118,551 reduced a CPP expression long-term (Fitzgerald et al., 2016).

Herein, we investigated the necessity of β-ARs in driving memory retrieval, retention, and acquisition/consolidation (learning) of cocaine associated memories in both male and female rats. Our behavioral results corresponded to previous findings showing that β-adrenergic receptor antagonists have immediate and long-lasting effects to decrease CPP memories in males. In females, Betaxolol transiently decreased CPP memories, whereas ICI 118,551 had long lasting, but not immediate effects to decrease CPP memories. Propranolol, however, impaired memory retrieval and retention in females. Additionally, ICI 118,551 and propranolol had immediate and persistent effects to decrease CPP memory expression. This could potentially indicate that β-ARs modulate cocaine-seeking behaviors.

## Data availability statement

The raw data supporting the conclusions of this article will be made available by the authors, without undue reservation.

## Ethics statement

The animal study was approved by University Committee for the Use and Care of Animals at the University of Mississippi. The study was conducted in accordance with the local legislation and institutional requirements.

## Author contributions

MB: Writing – review & editing, Writing – original draft, Methodology, Investigation, Formal analysis, Data curation. BM: Writing – review & editing, Writing – original draft, Visualization, Validation, Software, Investigation. SK: Writing – review & editing, Writing – original draft, Validation, Supervision, Project administration, Methodology, Investigation. CC: Writing – review & editing, Writing – original draft, Investigation. AK: Writing – review & editing, Writing – original draft, Visualization, Supervision, Resources, Project administration, Methodology, Funding acquisition, Formal analysis, Data curation, Conceptualization.
